# Impact of Web-Based Meeting Platform Usage on Overall Well-Being among Higher Education Employees

**DOI:** 10.3390/ejihpe11020028

**Published:** 2021-04-19

**Authors:** Martha E. Kershaw, Shannon P. Lupien, Jennifer L. Scheid

**Affiliations:** 1 Department of Nursing, Daemen College, Amherst, NY 14226, USA; 2 Department of Psychological Sciences, Daemen College, Amherst, NY 14226, USA; slupien@daemen.edu; 3 Departments’ of Health Promotion and Physical Therapy, Daemen College, Amherst, NY 14226, USA; jscheid@daemen.edu

**Keywords:** meeting fatigue, web-based meeting platform, pandemic, well-being

## Abstract

During the ongoing global pandemic, faculty, staff and administrators at colleges and universities experienced an increase in meetings using web-based platforms. Challenges were identified related to the changes from face-to-face to web-based meetings, including internet connectivity, inadequate technology and distractions in the online environment, which led to questions about how meetings that use web-based platforms may contribute to overall stress and well-being during the pandemic. The research related to the use of web-based meeting platforms is limited. However, some anecdotal evidence suggests that impacts from web-based meeting platforms could include frustration, sleep issues and fatigue, which contribute to overall well-being. The purpose of this study was to determine if a relationship exists between a number of potentially related web-based meeting factors including the frequency and length of the meetings and comfort level with the platform and overall well-being. This study involved (*N* = 164) male, female and nonbinary participants over 18 years of age who worked as tenured, tenure-track, or nontenure track faculty, staff and administrators at colleges/universities in the United States during the global pandemic. The participants were recruited via both social media and email and were provided with a link to the survey tool, which included demographic and web-based meeting questions (e.g., frequency, length, and comfort) along with scales to measure perceived stress, subjective well-being, mental fatigue and sleep quality. The current study did not find a relationship between the frequency of meetings and overall well-being (*p* = 0.294). However, statistically significant relationships were found between meeting length and overall well-being (*p* = 0.003) and between comfort with the web-based meeting platform and overall well-being (*p* = 0.030). Based on the findings of this study, meeting organizers may consider scheduling meetings for less than two hours and providing training to ensure participants are proficient in the web-based meeting platform in order to support overall well-being.

## 1. Introduction

With the global pandemic related to novel coronavirus that began in the spring semester of 2020, which led to subsequent pause, stay at home, or quarantine orders, colleges and universities in the United States moved operations to a remote setting, including teaching, student support, and meetings. In an effort to continue to have face-to-face options for communication while remote, meetings among faculty, staff and administrators and meetings with students were conducted via web-based meeting platforms. The web-based platforms involve logging in from remote locations with participants live and often visible via computer/smart device camera and microphone.

The switch from in-person, face-to-face meetings to remote meetings using a web-based meeting platform creates a number of challenges. In the remote setting, meetings may have been more frequent because of the rapid changes occurring on college and university campuses. Additionally, there were multiple uses for the communication and variability in the time needed to get through items that may normally have been quickly discussed within offices or when a student stopped by during office hours. Communication via a web-based meeting platform is often planned ahead of time and more sedentary whereas face-to-face communication on campus could be more spontaneous and active, including occurring during a walk across campus or in the hallway between classes. Communication also may be impacted by poor internet quality, inadequate technology, or several types of distractions within the remote environment. Furthermore, faculty, staff, and administrators may also be impacted by general pandemic-related stress. They may be responsible for their children’s education, caring for a family member who contracted the virus, be fighting the virus themselves, or even have had a change in financial situation or other loss as a result of this pandemic.

It is important to understand how meetings using web-based platforms may contribute to overall stress and well-being during this difficult time. However, the research related to web-based meeting platforms and their impact on overall well-being is limited. Anecdotal literature has used the term “Zoom Fatigue” in the context of meetings using web-based platforms. Wiederhold [1] identifies that online communication has an intrinsic delay, so face-to-face communication using a web-based meeting platform is not in real-time, but rather has a slight delay. The participants expect real-time responses and experience mental fatigue from attempting to re-establish real-time communication. Bailenson [2] identifies that participants are limited to a small space where they can be visible in a Zoom meeting, thereby limiting the mobility of the participants.

Wiederhold [1] and Bailenson [2] explain that the nonverbal communication that can be picked up in person is missing when using web-based meeting platforms. Weinderhold [1] includes that only the face is available for the speaker to view rather than the whole body like in a face-to-face meeting. It is difficult to know if meeting participants are understanding the points being made, as the speaker cannot read the body language that is normally available during a face-to-face meeting. The speaker must spend additional time preparing clarifying information to ensure points are made effectively. Bailenson [2] identifies that speakers need to work harder to be clear in communication without nonverbal cues. Baker and Murphy [3] identify that preparation for online meetings may take longer in order to make sure the information is clear. Bailenson [2] indicates that maintaining prolonged eye contact in a meeting using Zoom is unusual and creates an intensity that is unusual for work colleagues. In a face-to-face meeting, attendees would not stare directly at the speaker for the whole time they are speaking, but on Zoom, the direct gaze is often nonstop. Additionally, Nadler [4] includes that stress can occur if the platform does not function correctly or the internet connection is unstable. Although this research on Zoom fatigue is largely anecdotal, some empirical research exists examining face-to-face meeting fatigue that may also be relevant for web-based meeting platforms.

Luong and Rogelberg [5] found that among administrative employees working in a university setting, face-to-face meeting load had a negative relationship with employee daily well-being. The meeting load included the frequency of meetings and the length of time spent in meetings together. The results were limited to daily well-being and did not account for the compounding of the meeting load over time. In the current study, we examine overall well-being related to meetings that use a web-based platform. As past research showed that the frequency and length of the meetings were important factors for well-being, these factors should also apply to remote meetings using web-based meeting platforms. Thus, the current study will build upon the in-person meeting research and expand it to web-based meeting platforms and include additional factors, such as frequency, length and comfort with the web-based meeting platform, and how they relate to measures of overall well-being, such as perceived stress, mental fatigue, sleep quality, and subjective well-being. Other factors were considered in this study as potential contributing factors to stress and overall well-being, including the gender and the role of the participant, the number of school age children the participant had, any physical activity the participant was involved in, and any major stressful events the participant may have experienced in addition to the pandemic.

The purpose of the current study was to determine if a relationship exists between a number of potentially related factors, including the frequency and length of meetings using a web-based meeting platform along with comfort level in using the platform and the overall well-being of faculty, staff, and administrators at colleges/universities in the United States. We hypothesized that staff, faculty and administrators who had more frequent meetings using a web-based meeting platform would report lower overall well-being. Additionally, we hypothesized that staff, faculty and administrators who had longer meetings using a web-based meeting platform would indicate lower overall well-being. Lastly, we hypothesized that staff, faculty and administrators who had greater comfort with the web-based meeting platform they were using would have a higher overall well-being.

## 2. Materials and Methods

### 2.1. Participants

Participants consisted of male, female, and nonbinary individuals over 18 years of age who worked as tenured, tenure-track, or nontenure track faculty, staff and administrators at colleges/universities in the United States. The participants had been impacted by changes that occurred to their institutions during the global pandemic. Participants were recruited via social media (Facebook, Instagram, Twitter, and LinkedIn) using a flyer which explained inclusion criteria (18 years of age and working as faculty, staff or administrator at a college or university) and the survey link. Additionally, participants were recruited via email initially at a small regional private college in Western New York and then shared via email to colleagues across the United States. Participants accessed the study using a link to the survey tool which included demographic and technology information along with Cohen et al. [6] Perceived Stress Scale, Topp et al. [7] The World Health Organization-Five Well-Being Index (WHO-5), Johansson et al. [8] Mental Fatigue Scale and Buysse et al. [9] Pittsburgh Sleep Quality Index (PSQI). The survey was open from June 2020 through December 2020. The college’s institutional review board approved the study (Daemen College Human Subject Research Review Committee, HP.0120.X.034, 21 May 2020), and informed consent was obtained from all the subjects via the survey tool prior to study participation.

### 2.2. Data Collection

#### 2.2.1. Technology Information

The technology section of the survey asked participants for information their use of web-based meeting platforms. Frequency was measured by participant reports of how often they used a web-based meeting platform in a week. Response options consisted of “less than once a week”, “once a week”, “2–4 times per week”, “once a day”, “2–4 times per day”, “more than 4 times per day” and an “other” category for participants to specify their frequency of use. Length was measured by the participant report of the average length of meetings using the web-based meeting platform. This included ranges of time from “under one hour”, “one hour”, “two hours”, “three hours”, “more than three hours” and an “other” category for participants to specify their length of meetings. Comfort was measured as the participant-reported comfort level with the web-based meeting platform that they use most often. The responses included “I consider myself proficient”, “I am able to use the web based meeting platform without help”, “I often have questions”, “I need help every time” and “other” where the participant could specify their comfort level.

#### 2.2.2. Stress

Stress was measured using Cohen et al. [6] Perceived Stress Scale. The Perceived Stress Scale (PSS) is a 10-item questionnaire that asks participants about their feelings and thoughts during the last month. While the scale asks participants to consider their answers based on the last month, the researchers asked the participants to consider their answers from the beginning of the pandemic. This was initially phased as “since the beginning of April” and later expanded to “during the pandemic”. Examples of the questions on the PSS include: “How often have you been upset because of something that happened unexpectedly?”, “How often have you felt that things were going your way?”, “How often have you felt that you were on top of things?”, and “How often have you felt difficulties were piling up so high that you could not overcome them?”. The 10 items were rated on a 5-point scale from 0 (never) to 4 (Very Often), with the 4 positive items reversed scored and then taking the sum of all 10 items. Higher scores indicated higher perceived stress.

#### 2.2.3. Well-Being

Basic well-being was measured using the World Health Organization-Five [7] Well-Being Index (WHO-5). The WHO-5 includes a five-question survey that asks participants to rate five statements related to how they have been feeling over the last two weeks using a 0–5 scale (0 = at no time, 1 = some of the time, 2 = less than half the time, 3 = more than half the time, 4 = most of the time, 5 = all of the time) with higher numbers indicating higher well-being. Examples of the questions on the WHO-5 include: “I have felt cheerful and in good spirits”, “I have felt active and vigorous, and I woke up feeling fresh and rested”. Scores for well-being were obtained by summing the 5 responses and multiplied by 4. The results can range from 0 to 100 with 0 indicating the lowest well-being score and 100 indicating the highest.

#### 2.2.4. Mental Fatigue

Mental Fatigue was measured using Johansson et al. [8] Mental Fatigue Scale. The Mental Fatigue Scale (MFS) is a 15-question self-assessment scale, which asks the participant to respond to how they have felt during the past month. While the scale asks participants to consider their answers based on the last month, the researchers asked the participants to consider their answers from the beginning of the pandemic. This was initially phrased as “since the beginning of April” and later expanded to “during the pandemic.” Participants were asked to select the statement that best describes their problems based on four statements that describe: No (0), Slight (1), Fairly serious (2) and Serious (3) problems. Questions on the MFS focus on fatigue, lack of initiative, mental fatigue, mental recovery, concentration difficulties, memory problems, slowness of thinking, sensitivity to stress, increased tendency to become emotional, irritability or a “short fuse”, sensitivity to light, sensitivity to noise, decreased sleep at night, increased sleep, 24-h variations. The sum of the responses was obtained, and a score above 10 is seen to indicate a problem with mental fatigue.

#### 2.2.5. Sleep Quality

Sleep was measured using Buysse et al. [9] Pittsburgh Sleep Quality Index. The Pittsburgh Sleep Quality Index (PSQI) asks participants to consider their usual sleep habits during the past month only. The researchers expanded this to “during the pandemic” and directed participants that their answers should indicate the most accurate reply for the majority of days and nights during the pandemic. The initial questions in the questionnaire include: “When did you usually go to bed?”, “How long (in minutes) did it take you to fall asleep each night?”, “What time did you usually get up in the morning?”, “How many hours of sleep did you get at night?” and “How many hours were you in bed?”. One question inquired about trouble sleeping, asking participants to rate their responses on a scale of 0 (positive) to 3 (extremely negative) to examples including: “cannot get to sleep within 30 min”, “cannot breathe comfortably”, “feel too hot and have bad dreams”. Additional questions using the same 0-3 scale included: “How often have you taken medicine (prescribed or over the counter) to help you sleep?”, “How often have you had trouble staying awake while driving, eating meals, or engaging in social activity?”, and “How much of a problem has it been for you to keep up enthusiasm to get things done?”. The final question asks, “how would you rate your sleep quality overall” using a scale of 0 (Very good) to 3 (Very bad). The responses were summed using a specific scoring calculation. A sum of 5 or greater indicates poor sleep quality. Permission to use the PSQI was obtained from University of Pittsburgh—of the Commonwealth System of Higher Education.

#### 2.2.6. Data Analysis

A one-way ANOVA was conducted to compare the effects of the predictor variables (frequency, length and comfort) individually on the outcome variable of overall well-being for participants. For each variable, response categories with less than 5 participants indicating a given response were combined into the next related category. Specifically, for frequency, response categories indicating “once a week” and “less than once a week” were combined into a single category “once a week or less”. For length, response categories indicating “two hours”, “three hours” and “more than three hours” were combined into one category of “two or more hours”. Lastly, for comfort with web-based meeting platform, response categories indicating “I often have questions” and “I need help every time” were also combined into a single category.

Overall well-being was calculated as a composite score of each of the individual predictor variables including stress, subjective well-being, mental fatigue and sleep quality. Each component of the composite was significantly correlated with the other components, and each led to a similar and expected pattern of results. Due to this, the predictor variables were combined into a composite of overall well-being to give a more complete picture of both physical and psychological well-being. To create the composite, Z scores were first calculated individually for each of the main predictor variables (i.e., perceived stress measured by Cohen et al. [6] Perceived Stress Scale, well-being measured by Topp et al. [7] World Health Organization-Five Well-Being Index (WHO-5), Johansson et al. [8] Mental Fatigue Scale, and the Buysse et al. [9] Pittsburgh Sleep Quality Index). The Z scores for stress, mental fatigue, and sleep quality were then transformed by multiplying by -1 so that higher numbers of each predictor variable were all associated with higher well-being. Next, the composite variable was created by calculating the average of the Z score version of the four predictor variables.

We also explored if there were any significant relationships between participant demographic factors and the variables of interest. We found that age and gender were significantly related to overall well-being. Specifically, the younger the participant, the lower their overall well-being [F(2; 161) = 13.669, *p* = 0.000]. Additionally, females reported lower overall well-being than males and the one nonbinary participant [F(2; 161) = 10.019, *p* = 0.000]. Thus, covariates of age and gender were included in the data analyses described below. All data were analyzed using the SPSS for Windows (version 26.0, Chicago, IL, USA) statistical software package.

## 3. Results

The sample (*N* = 164) included 90 faculty members (Tenured Faculty, 42; Tenure Track Faculty 26; Non tenure track faculty 22), 31 staff members, 20 administrators, and 23 others/preferred not to answer. The sample included 140 females, 23 males and 1 nonbinary/fluid participant. The reported age range of participants is indicated by the following: less than 18 (*n* = 0), 18–24 (*n* = 3), 25–34 (*n* = 32), 35–44 (*n* = 51), 45–54 (*n* = 43), 55–64 (*n* = 28), and 65–74 (*n* = 7). Additionally, participants reported using the following web-based meeting platforms most frequently: Zoom (*n* = 121), Google Meets (*n* = 10), Google Hangouts (*n* = 3), GoToMeeting (*n* = 1), and other (*n* = 29). The uses of the web-based meeting platforms included: committee meetings (*n* = 133), student advisement (*n* = 90), office hours (*n* = 88) and synchronous class meetings (*n* = 82).

### 3.1. Meeting Frequency

A one-way ANOVA was conducted to compare the effect of the frequency of meetings using a web-based meeting platform in a week and the overall well-being of participants. We hypothesized that staff, faculty and administrators who had more frequent meetings using a web-based meeting platform would have lower overall well-being. However, there was not a significant effect of frequency on overall well-being at the *p* < 0.05 level [F(4, 151) = 1.245, *p* = 0.294].

### 3.2. Meeting Length

**Figure 1:**
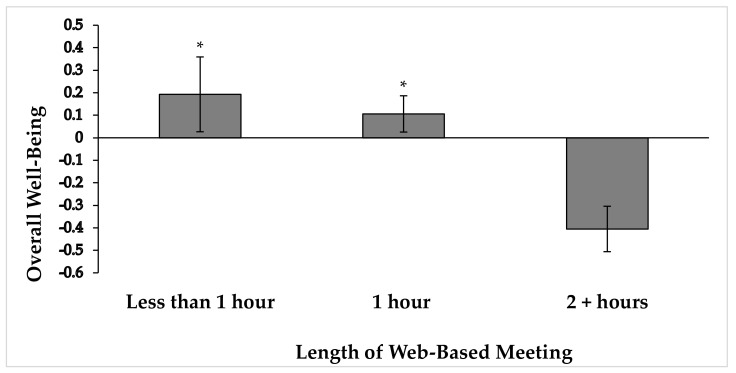
Overall well-being composite score (mean ± standard error of the mean) grouped by average length of meeting when using a web-based platform. * *p* < 0.001 when compared to meetings lasting two hours or longer (2 + hours).

A one-way ANOVA was conducted to compare the effect of length of meeting using a web-based meeting platform and the overall well-being of the participants. We hypothesized that staff, faculty and administrators who had longer meetings using a web-based meeting platform would have lower overall well-being. The analysis was significant at the *p* < 0.01 level [F(2; 154) = 7.028, *p* = 0.001, η_p_^2^ = 0.084], such that longer meetings indicated lower well-being. Specifically, pairwise comparisons indicated that meetings lasting two hours or longer (M = −0.405, SD = 0.583) were related to significantly lower well-being than meetings lasting both one hour (M = 0.106, SD = 0.834, *p* = 0.000) and less than one hour (M = 0.193, SD = 0.762, *p* = 0.005). However, meetings lasting one hour and less than one hour did not significantly differ in their relationship to overall well-being (*p* = 0.772) (See Figure 1).

### 3.3. Comfort Level with Web-Based Meeting Platform

**Figure 2:**
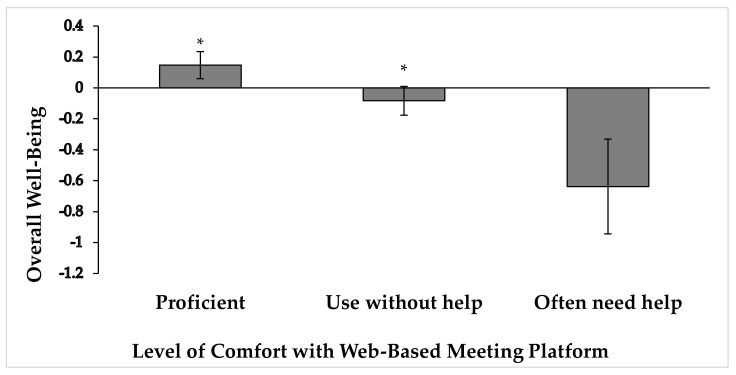
Overall well-being composite score (mean ± standard error of the mean) grouped by comfort using a web-based platform. * *p* < 0.06 when compared to often needing help, i.e., being able to use the meeting platform without help and often having questions/needing help every time.

A one-way ANOVA was conducted to compare the effect of comfort level using a web-based meeting platform and the overall well-being of the participants. We hypothesized that staff, faculty and administrators who had greater comfort with the web-based meeting platform they were using would have a higher overall well-being. There was a significant effect of comfort on overall well-being at the *p* < 0.05 level [F(2; 158) = 3.587, *p* = 0.030], such that greater comfort was related to higher well-being. Specifically, participants who identified themselves as proficient experienced higher levels of well-being (M = 0.147, SD = 0.812) compared to participants who indicated that they could use the meeting platform without help (M = −0.084, SD = 0.795, *p* = 0.055) and that they often had questions or needed help every time (M = −0.638, SD = 0.613, *p* = 0.033). However, the participants able to use the meeting platform without help and often having questions/needing help every time did not significantly differ in their relationship to overall well-being (*p* = 0.123) (See Figure 2).

## 4. Discussion

This is the first study to specifically look at the relationship between the use of web-based meeting platforms and overall well-being in faculty, staff and administrators in colleges and universities in the United States. We found evidence of a relationship between both the length of the meeting and the comfort level with the web-based meeting platform and overall well-being. We also expected to find a relationship between the frequency of meetings using a web-based meeting platform and overall well-being, but the results did not support this hypothesis. Other factors considered in this study as potential contributing factors to stress and overall well-being including the role of the participant, the specific type of web-based meeting (e.g., class, office hours, committee meetings, etc.), the number of school age children the participant had, any physical activity the participant was involved in, and any major stressful events the participant may have experienced in addition to the pandemic. No relationships were found between these variables and overall well-being.

The global pandemic related to novel coronavirus, which began in the spring semester of 2020, moved operations on college and university campuses in the United States to a remote setting, including teaching, student support, and meetings. Luong and Rogelberg [5] found that face-to-face meeting load in administrative employees in the university setting had a negative relationship with employee daily well-being. Meeting load included the frequency of meetings along with time spent in meetings. When looking at meetings involving web-based meeting platforms, frequency and length may also have a relationship with overall well-being. Additionally, comfort level with the web-based meeting platform could also impact overall well-being.

While we examined relationships between the frequency and length of meetings using a web-based meeting platform along with comfort level with the platform and overall well-being, we did not find a relationship between the frequency of meetings and overall well-being. There was a relationship found between the length of meetings using a web-based platform and overall well-being, specifically that meetings one hour or less were related to higher well-being compared to those lasting two hours or longer. This may be related to Wiederhold’s [1] assertion of an intrinsic delay in online communication so people in the meeting may feel disconnected. Bailenson [2] and Baker and Murphy [3] identified that there is also the potential difficulty in reading nonverbal communication and thus assessing if the information is being understood, necessitating extra work to ensure clarity. Baker and Murphy [3] also suggested that meetings should be approximately 40 to 45 min, which is supported by the finding that shorter meetings were associated with higher well-being.

Cranford [10] identified that with the increase in remote working due to the pandemic, employees are not able to drop by and ask a question of a peer, but instead need to schedule a web-based meeting, thus increasing the frequency of meetings. While meeting frequency was not found to impact overall well-being in the current study, the meetings are likely more structured, which would require the hyper focus that Cranford [10] identified as being draining. Maintaining that hyper focus for longer periods of time may have contributed to the current findings that shorter meetings were associated with higher well-being.

There also was a relationship between comfort level with the web-based meeting platform being used and overall well-being, specifically that those who considered themselves to be proficient in using the web-based meeting platform indicated higher well-being than those who could use the platform without help or those who often had questions or needed help every time. The comfort level associated with being proficient may decrease the stress that Nadler [4] identified can occur if the platform does not function properly or the internet connection is unstable. To prevent or address potential technical issues, Baker and Murphy [3] suggest participants should sign in prior to the start of the meeting.

Prepandemic, Page and Vella Brodrick [11] identified a model of well-being that included a connection between well-being and the overall health of an organization. In their study, well-being was related to the retention of employees, which is an important consideration for colleges and universities postpandemic. Changes driven by the results of the current study, such as limiting meeting length and facilitating comfort with the web-based meeting platform, should improve well-being which could, in turn, lead to better retention and thus a healthier organization. Additionally, prepandemic, Bakker and Demerouti [12] used the Job Demands–Resources theory to discuss employee well-being, which indicates that is important to identify the demands and resources that could impact employees. The leaders of organizations can improve processes to support employee well-being. Regarding the use of web-based meetings, one process that could be suggested for an organization is to implement a meeting-length limit of one hour, which could positively impact employee well-being by decreasing demands. Additionally, providing easily assessable training to promote comfort with the web-based meeting platform could increase resources, and additionally impact well-being. Meyer and Hünefeld [13] examined employee well-being and cognitive demands including a variable called “facing new tasks”. Their findings indicate that there is a relationship between the cognitive load created by facing new tasks and employee well-being and that facing new tasks could be a stressor. The addition of using web-based meeting platforms could be considered a new task for many. Providing classes or training to support the use of this new tool could potentially decrease or eliminate the stressor and ultimately decrease cognitive load, leading to improved well-being.

Overall, the results of the current study have important implications for faculty, staff, and administrators within the higher education setting, not only during the ongoing global pandemic, but also likely going forward. Zackal [14] identifies the potential that some colleges and universities will continue to have a portion of their faculty, staff and administration working remotely beyond the pandemic. Meetings using web-based meeting platforms will therefore likely continue to be a necessary tool. Meeting length should be considered by these institutions to promote well-being in their employees. Specifically, efforts could be made so that meetings last no more than an hour. Additionally, offering classes or training to promote comfort with the web-based meeting platform would also support well-being in faculty, staff and administration working remotely. Furthermore, the implications of the current study likely extend beyond the higher education setting. With the use of web-based meeting platforms in order to continue day-to-day operations in many employment settings, the findings of this study may have implications in other areas like medicine. Moore et al. [15] identified that telehealth will have uses beyond the pandemic. While visits with providers are often limited, the findings related to the length of meetings might be useful in planning the number of online visits a provider completes in a row. The level of comfort with web-based platforms could also translate to a platform used for telehealth visits, and thus having tutorials available for both patient and provider could likely support well-being in the healthcare field as well.

There are strengths to the current study including addressing the empirical relationship between meetings using a web-based meeting platform and overall well-being. Currently, only anecdotal evidence related to the impact of the use of web-based meeting platforms was found in the literature. In addition to the current study, we encourage other studies to further explore the impact of the use of web-based meeting platforms to address the claims made in the anecdotal literature.

This current study had limitations including the sample size, the time that the study was launched and the length of survey. Due to the sample size the information may not be completely generalizable. However, the participants were fairly diverse in their representation of different types of faculty, staff, and administrators working in several different colleges and universities across the country. Future studies with a larger sample size could help provide additional support for the results of this study. The size of the sample may have been impacted by the timing of the study launch and the length of the survey. The study was launched in June 2020 as the spring semester ended and the global pandemic appeared to be lasting longer than anticipated. Future studies should be launched during the semester rather than at the end. The end of the semester tends to be a busy time for faculty so requests for research participation may be set aside at that time. The original study consisted of approximately 93 questions and took about 38 min to complete. Future research should focus specifically on the frequency and length of and comfort with meetings using web-based meeting platforms and overall well-being outside of the global pandemic. It would also be interesting to repeat the study focusing on face-to-face meetings to see if the patterns are similar.

In conclusion, based on the findings of this study, meeting length is an important consideration. As having meetings one hours or less was related to higher well-being, organizers might consider scheduling shorter meetings. However, because the frequency of meetings using a web-based meeting platform was not related to well-being in this study, more frequent meetings in lieu of longer meetings might be possible without significantly impacting well-being. Another consideration when planning a web-based meeting would be to offer training for the participants to help increase the comfort level and increase proficiency, which could also be beneficial for overall well-being as demonstrated in the current study.

## Data Availability

The data presented in this study are available on request from the corresponding author.
